# PEG‐23 glyceryl distearate, a multifunctional skin‐supporting material, upregulates the expression of factors associated with epidermal barrier and hydration

**DOI:** 10.1111/ics.70005

**Published:** 2025-07-10

**Authors:** Tatsuro Miyoshi, Brian C. Keller, Sui Nakagawa, Takashi Ashino, Satoshi Numazawa

**Affiliations:** ^1^ Beverly Glen Laboratories, Inc. Newport Beach California USA; ^2^ Department of Toxicology Showa Medical University Graduate School of Pharmacy Shinagawa Tokyo Japan

**Keywords:** barrier function, epidermal keratinocyte, GDS‐23, hydration, multifunctional DDS material, skin health

## Abstract

**Objective:**

As the outermost organ of the human body, the skin plays a critical role in protecting against external agents and oxidative stress, as well as in preventing excessive water loss. Among its layers, the stratum corneum of the epidermis is central to both barrier function and moisture retention, with its structural integrity significantly influencing skin health and appearance. Polyethylene glycol‐23 glyceryl distearate (GDS‐23) can form niosomes, liposome‐like structures with multiple layers and serve as a carrier in drug delivery systems (DDS). We previously reported that GDS‐23 exerts multiple biological effects, suppressing inflammation and enhancing the endogenous antioxidant system via Nrf2 activation through phosphorylation of the autophagy adaptor protein p62. In this study, we investigated the effects of GDS‐23 on epidermal barrier function and moisture retention using normal human epidermal keratinocytes (NHEKs) and a three‐dimensional (3D) epidermal model, mimicking human skin structure.

**Methods:**

NHEKs and the 3D epidermal model were treated with GDS‐23. Gene expression in NHEKs was analysed using real‐time polymerase chain reaction, while the levels of epidermal barrier‐ and moisture retention‐related factors in the 3D model were evaluated using immunofluorescence staining.

**Results:**

Treatment with GDS‐23 upregulated the mRNA expression of genes involved in stratum cornea formation (filaggrin and loricrin), moisture retention (aquaporin 3 and hyaluronan synthase 3) and intercellular lipid synthesis (ceramide synthase, sulfotransferase 2B1 and peroxisome proliferator‐activated receptor α) in NHEKs. Additionally, treatment with K67, which inhibits p62 phosphorylation and thereby suppresses Nrf2 activation via a non‐canonical mechanism, suppressed the GDS‐23‐induced expression of filaggrin, loricrin, ceramide synthase, sulfotransferase 2B1 and aquaporin 3. In the 3D epidermal model, GDS‐23 treatment upregulated the expression of Nrf2, downstream antioxidant factors and proteins involved in stratum corneum formation and moisture regulation. Mechanistically, GDS‐23 enhanced endogenous antioxidant function and modulated the expression of molecular markers associated with epidermal barrier and moisture retention, thereby potentially contributing to skin homeostasis.

**Conclusion:**

As GDS‐23 contributes to the maintenance of skin homeostasis, it is expected to have future applications in the cosmetic field and in the treatment of skin disorders. Overall, GDS‐23 holds promise as a “multifunctional DDS material” that promotes skin health.

## Graphical abstract

Polyethylene glycol‐23 glyceryl distearate (GDS‐23) forms niosomes and penetrates the skin. Our results suggest that the pharmacological effects following dermal penetration of GDS‐23 may enhance skin homeostasis by improving barrier function and moisture retention.

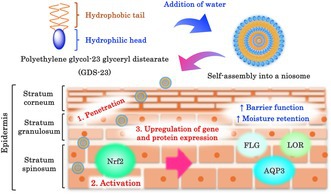

## Résumé

**Objectif:**

En tant qu'organe le plus externe de l'organisme, la peau joue un rôle essentiel dans la protection contre les agents externes et le stress oxydatif, ainsi que dans la prévention de la perte excessive d'eau. Parmi ses couches, la couche cornée de l'épiderme est essentielle à la fois à la fonction barrière et à la rétention de l'humidité, son intégrité structurelle influençant considérablement la santé et l'apparence de la peau. Le distéarate de glycéryle polyéthylène glycol‐23 (GDS‐23) peut former des niosomes, des structures semblables à des liposomes à plusieurs couches, et servir de vecteur dans les systèmes d'administration de médicaments. Nous avons précédemment rapporté que le GDS‐23 exerce de multiples effets biologiques, supprimant l'inflammation et renforçant le système antioxydant endogène via l'activation de Nrf2 par la phosphorylation de la protéine adaptatrice de l'autophagie p62. Dans cette étude, nous avons étudié les effets du GDS‐23 sur la fonction barrière épidermique et la rétention de l'humidité à l'aide de kératinocytes épidermiques humains normaux (NHEK) et d'un modèle épidermique tridimensionnel (3D) imitant la structure de la peau humaine.

**Méthodes:**

Les NHEK et le modèle épidermique 3D ont été traités avec du GDS‐23. L'expression génique dans les NHEK a été analysée à l'aide d'une réaction en chaîne par polymérase en temps réel, tandis que les niveaux de facteurs liés à la barrière épidermique et à la rétention de l'humidité dans le modèle 3D ont été évalués à l'aide d'une coloration par immunofluorescence.

**Résultats:**

Le traitement par GDS‐23 a régulé à la hausse l'expression de l'ARNm des gènes impliqués dans la formation de la couche cornée (filaggrine et loricrine), la rétention de l'humidité (aquaporine 3 et hyaluronane synthase 3) et la synthèse des lipides intercellulaire (céramide synthase, sulfotransférase 2B1 et récepteur α activé par les proliférateurs de peroxysome) dans les NHKE. De plus, le traitement par K67, qui inhibe la phosphorylation de p62 et supprime ainsi l'activation de Nrf2 via un mécanisme non canonique, a supprimé l'expression induite par GDS‐23 de la filaggrine, de la loricrine, de la céramide synthase, de la sulfotransférase 2B1 et de l'aquaporine 3. Dans le modèle épidermique 3D, le traitement par GDS‐23 a régulé à la hausse l'expression de Nrf2, des facteurs antioxydants en aval et des protéines impliquées dans la formation de la couche cornée et la régulation de l'humidité. Sur le plan mécanique, le GDS‐23 a amélioré la fonction antioxydante endogène et a modulé l'expression des marqueurs moléculaires associés à la barrière épidermique et à la rétention de l'humidité, contribuant ainsi potentiellement à l'homéostasie cutanée.

**Conclusion:**

Étant donné que le GDS‐23 contribue au maintien de l'homéostasie cutanée, il devrait trouver des applications futures dans le domaine cosmétique et dans le traitement des troubles cutanés. Dans l'ensemble, le GDS‐23 est prometteur en tant que « matériau multifonctionnel pour systèmes d'administration de médicaments » qui favorise la santé de la peau.

## INTRODUCTION

**FIGURE 1:**
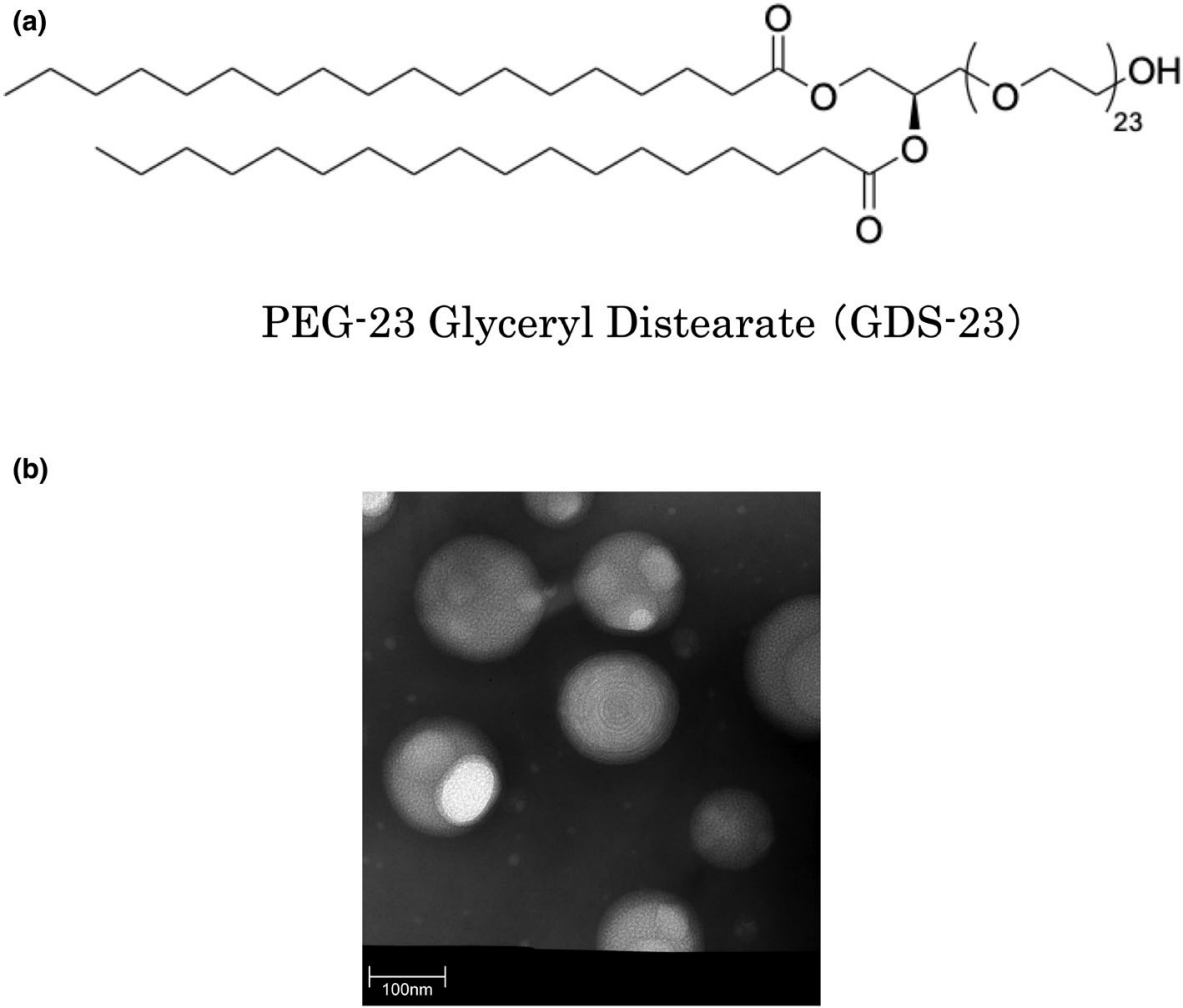
(a) Typical structural formula of the diacylglycerol polyethylene glycol adduct. The structure of polyethylene glycol‐23 glyceryl distearate (GDS‐23), which was used in this study, is presented. (b) Transmission electron microscopy (TEM) image of niosomes composed of the diacylglycerol polyethylene glycol adduct.

Diacylglycerol polyethylene glycol (PEG) adducts (Figure 1a) are non‐ionic surfactants capable of easily forming niosomes (Figure 1b), which can form vesicles with multiple layers [1]. Niosomes are nano‐sized vesicles composed of non‐ionic surfactants, and they have a liposome‐like structure made of phospholipids. Compared to liposomes, niosomes offer advantages such as a simple preparation process, high cost efficiency and great stability, making them attractive for various applications, including cosmetic use [2, 3]. Furthermore, owing to their amphiphilic bilayer structure, niosomes can encapsulate both hydrophilic and lipophilic compounds, making them useful not only in drug delivery systems (DDS) but also for the delivery of functional ingredients into the skin [4, 5].

A previous study examined the DDS applications of niosomes, especially the ability of the non‐ionic surfactant “diacylglycerol PEG adducts” to form niosomes and enhance the skin permeability of drugs [6, 7]. Diacylglycerol PEG adducts not only aid in drug transport but also exhibit anti‐inflammatory effects by inhibiting phospholipase A and cyclooxygenase‐2 activities [8]. Mechanistically, diacylglycerol PEG adducts activate Nrf2 through a non‐canonical mechanism mediated by p62 phosphorylation, thereby enhancing the endogenous antioxidant system in human epidermal keratinocytes [9]. Proper activation of Nrf2 promotes skin barrier function and regulates the inflammatory response, together contributing to the maintenance of skin homeostasis [10].

As the outermost barrier of the human body, the skin plays a crucial role in protecting against external substances, microbial invasion, oxidative stress and excessive moisture loss [11]. The epidermis and stratum corneum, the outermost layers of the skin tissue, are central to barrier and moisture‐retention functions, with the state of the stratum corneum considerably influencing these functions [12]. In addition to maintaining skin health, the condition of the stratum corneum is critical from an aesthetic perspective, and it has become a key target in cosmetic product development [13, 14]. The stratum corneum primarily consists of corneocytes, intercellular lipids and natural moisturizing factors (NMFs). Maintaining appropriate moisture levels in epidermal cells and the stratum corneum is crucial to skin homeostasis, with hygroscopic NMFs being partially responsible for moisture retention in the stratum corneum [15]. NMFs include free amino acids and the degradation products of filaggrin (FLG), generated through the enzymatic breakdown of its precursor profilaggrin by enzymes such as caspase‐14 and bleomycin hydrolase (BH) during keratinocyte differentiation [16, 17]. Additionally, intercellular lipids, mainly composed of ceramides, free fatty acids, cholesterol and cholesterol sulfate, form a lamellar structure on the cornified envelope (CE) at the periphery of stratum corneum cells, filling the intercellular spaces and creating a barrier that prevents the entry of external substances and transepidermal water loss. The CE is primarily composed of hydrophobic proteins such as loricrin (LOR) and involucrin, with LOR forming strong cross‐linking bonds through transglutaminases, leading to a robust and mature CE that stabilizes the lamellar structure of intercellular lipids [11, 17, 18, 19]. Mature CE not only forms a robust structure through protein cross‐linking but also plays a crucial role in moisture‐retention functions through its hydrophobic nature and by stabilizing the lamellar structure of intercellular lipids [20].

The epidermis is characterized by the presence of hyaluronic acid (a glycosaminoglycan), which is synthesized by hyaluronic acid synthase 3 (HAS3) and has a unique ability to bind and retain water molecules [21, 22]. Additionally, aquaporin 3 (AQP3), a member of the membrane‐spanning protein family that facilitates the flow of water across cell membranes, regulates epidermal water transport and maintains skin tissue homeostasis [23]. In the epidermis, AQP3 functions primarily in water level regulation, allowing epidermal cells to absorb water and swell, owing to their permeability to water and solutes such as glycerol and urea [24]. Moreover, peroxisome proliferator‐activated receptor α (PPARα), which is involved in lipid metabolism in the liver, is expressed in the epidermis, contributing to the maintenance of the epidermal barrier function by inducing keratinocyte differentiation, promoting lipid production and suppressing skin inflammation [25, 26].

Research suggests that Nrf2 activation exerts positive effects against various skin diseases by contributing to the maintenance of the epidermal barrier and moisture retention [27, 28]. Based on these findings, here, we investigated how a diacylglycerol PEG adduct affects skin barrier function and moisture retention and whether its effects are mediated through Nrf2 activation. We focused on the functional effects of the diacylglycerol PEG adduct, exploring potential applications in cosmetics and DDS.

## MATERIALS AND METHODS

### Reagents

Polyethylene glycol‐23 glyceryl distearate (GDS‐23; Figure 1) was obtained from J‐Network, Inc. (Newport Beach, CA, USA). ReverTra Ace qPCR RT Master Mix was purchased from Toyobo (Osaka, Japan). PowerUp SYBR Green Master Mix was procured from Thermo Fisher Scientific (Waltham, MA, USA). Goat anti‐rabbit IgG H&L (Alexa Fluor® 488) and goat anti‐mouse IgG H&L (Alexa Fluor® 488) were procured from Abcam (Cambridge, UK). Cellstain‐4′,6‐diamidino‐2‐phenylindole (DAPI) solution was obtained from Dojindo Laboratories (Kumamoto, Japan). K67 (2‐Acetonyl‐1,4‐bis[(4‐ethoxybenzensulfonyl)amino]naphthalene, N,N′‐(2‐(2‐oxopropyl)naphthalene‐1,4‐diyl)bis(4‐ethoxybenzenesulfonamide), N‐[2‐acetonyl‐4‐(4‐ethoxybenzenesulfonylamino)naphthalene‐1‐yl]‐4‐ethoxybenzenesulfonamide) were purchased from Sigma–Aldrich (St. Louis, MO, USA). Polymerase chain reaction (PCR) primer sets were purchased from Takara Bio (Shiga, Japan). The primer sets used and their sequences are presented in Table 1. Other reagents used were of analytical grade.

**TABLE 1:** Primers used in quantitative polymerase chain reaction.

Gene	Forward primer (5′–3′)	Reverse primer (5′–3′)
Glyceraldehyde‐3‐phosphate dehydrogenase (*GAPDH*)	GCACCGTCAAGGCTGAGAAC	TGGTGAAGACGCCAGTGGA
Filaggrin (*FLG*)	CAGTCACGTGGCAGTCCTCA	ACCATAGCTGCCATGTCTCCAA
Loricrin (*LOR*)	CCTACCTGGCCGTCCAAATA	GCAAACCTCGGGTAGCATCA
Hyaluronan synthase 3 (*HAS3*)	TCGGCGATTCGGTGGACTA	GGACTCGAAGCATCTCGATGG
Aquaporin 3 (*AQP3*)	GATCAAGCTGCCCATCTACACC	GCCATTGATCATATCCAAGTGTCC
Ceramide synthase 2 (*CerS2*)	TCTACGCCAAAGCCTCAGATCTC	GCTTGCCACTGGTCAGGTAGAA
Ceramide synthase 3 (*CerS3*)	GTGGCTGACATTTGGCTGGA	ACAATGAGGCGGCTGATGAA
Sulfotransferase family 2B member 1 (*SULT2B1*)	GGGACGACGACATCTTTATCATC	AGGCACCCACAATGGTCTCAC
Peroxisome proliferator activated receptor alpha (*PPARα*)	CGTATTAGAGGCCACCGATTTCA	CATTTCCAATTGCCAGCGTTC

### Cell culture

Normal human epidermal keratinocytes (NHEKs) were purchased from Kurabo (Tokyo, Japan) and cultured in HuMedia‐KG2 medium (Kurabo) at 37°C under 5% CO_2_ conditions. After detaching using 0.25% trypsin and 0.01% ethylenediaminetetraacetic acid disodium salt (EDTA‐2Na), the cells were reseeded on plates and cultured for subsequent experiments. Additionally, a three‐dimensional (3D) cultured epidermal model (LabCyte EPI‐MODEL 24) was purchased from Japan Tissue Engineering Co., Ltd. (Aichi, Japan) and cultured in the supplied assay medium at 37°C under 5% CO_2_ conditions.

### mRNA quantification

NHEKs were cultured in 96‐well plates (2.0 × 10^4^ cells/well) containing HuMedia‐KG2 medium for 24 h, followed by incubation in HuMedia‐KB2 containing GDS‐23 for an additional 24 h. For longer cultures exceeding 24 h, the cells were post‐cultured for 6 or 12 h in HuMedia‐KB2 alone. Cells in the control group were cultured in HuMedia‐KB2 alone for the specified durations. Total RNA was extracted from the cells using the RNeasy Mini kit (QIAGEN, Venlo, Netherlands), and then used for cDNA synthesis with a PCR Thermal Cycler Dice (Takara Bio). mRNA amplification and quantification were performed on the StepOne Real‐Time PCR System (Applied Biosystems; Thermo Fisher Scientific, Waltham, MA, USA) using PowerUp SYBR Green Master Mix and specific primers. Relative mRNA expression was calculated using the ΔΔCt method and normalized to the expression of *GAPDH* (an internal standard).

### mRNA quantification after treatment with the Nrf2 inhibitor

NHEKs were cultured in 96‐well plates (2.0 × 10^4^ cells/well) containing HuMedia‐KG2 medium for 8 h, followed by incubation in HuMedia‐KB2 containing K67 for 16 h and final incubation in HuMedia‐KB2 containing GDS‐23 for 24 h. For longer cultures exceeding 24 h, the cells were post‐cultured for 6 or 12 h in HuMedia‐KB2 alone. Cells in the control group were cultured in HuMedia‐KB2 alone for the specified durations. RNA extraction and cDNA synthesis were performed as described above. mRNA quantification was conducted using the ΔΔ*C*_t_ method, with *GAPDH* as the internal standard.

### Preparation of frozen sections from a 3D cultured epidermal model

Briefly, the 3D‐cultured epidermal model was acclimated overnight at 37°C in the provided assay medium. After acclimation, 50 μL of either 2% GDS‐23 aqueous solution (equivalent to approximately 12.2 mM) or purified water (as a control) was applied to the surface layers of the epidermal model, and the culture was continued for an additional 6 h. A treatment duration of 6 h was selected based on preliminary experiments, wherein although no cytotoxicity was observed with 24 h of exposure, the stratum corneum structure of the 3D epidermal model deteriorated under prolonged treatment. To adjust the pH of each sample to approximately 5.5, 0.02% citric acid and 0.08% trisodium citrate were added. The actual pH was 5.5 for the 2% GDS‐23 solution and 5.6 for the control. After 6 h of exposure, the samples were removed and washed, and the model was cultured for an additional 18 h without any application on the stratum corneum side.

For prolonged cultures exceeding 2 days, the medium was replaced every 2 days with fresh assay medium. At the designated time points, the epidermal model was embedded in optimal cutting temperature (OCT) compound, flash‐frozen in liquid nitrogen to create tissue cryoblocks and cut into 5‐μm‐thick frozen sections using a cryomicrotome (Cryostar NX70; Epredia, Portsmouth, NH, USA).

### Fluorescence immunostaining

The frozen tissue sections were mounted on glass slides, fixed with 4% paraformaldehyde for 5 min, washed with phosphate‐buffered saline without calcium and magnesium [PBS (−)] and blocked with 10% normal goat serum (dissolved in 3% bovine serum albumin (BSA)‐containing PBS [−]) for 1 h at room temperature (25 ± 3°C). After removing the blocking agent, the sections were incubated with primary antibodies diluted in 3% BSA/PBS solution overnight at 4°C. Thereafter, the sections were washed with PBS (−) and incubated in the dark with Alexa 488‐conjugated secondary antibodies diluted in 3% BSA/PBS solution for 1 h at room temperature. Finally, the sections were treated with 2.0 μg/mL DAPI in 3% BSA/PBS solution in the dark at room temperature for 10 min. After staining, the sections were washed with PBS (−) and observed under a fluorescence microscope (BZ‐X810; KEYENCE, Osaka, Japan). The dilution rates and origins of the primary antibodies were as follows: anti‐Nrf2 antibody (EP1808Y) (ab62352, rabbit monoclonal; Abcam) was used at a 100‐fold dilution, anti‐NAD(P)H‐quinone oxidoreductase (NQO1) antibody (EPR3309) (rabbit monoclonal, Abcam) was used at a 100‐fold dilution, anti‐heme oxygenase 1 (HO‐1) antibody (HO‐1‐1) (mouse monoclonal, Abcam) was used at a 200‐fold dilution, anti‐LOR antibody (EPR7149[2])‐C‐terminal (rabbit monoclonal, Abcam) was used at a 50‐fold dilution, anti‐FLG antibody (AKH1) (mouse monoclonal, Santa Cruz Biotechnology, Dallas, TX, USA) was used at a 50‐fold dilution, and an anti‐AQP3 antibody (F‐1) (mouse monoclonal, Santa Cruz Biotechnology) was used at a 50‐fold dilution. The secondary antibodies, Alexa Fluor® 488‐conjugated goat anti‐mouse IgG H&L (Abcam) and Alexa Fluor® 488‐conjugated goat anti‐rabbit IgG H&L (Abcam), were used at a 500‐fold dilution.

### Haematoxylin and eosin staining

Briefly, the frozen tissue sections were mounted on glass slides, fixed in 4% paraformaldehyde for 5 min, washed with purified water and stained using the Haematoxylin and Eosin (HE) Stain Kit (ScyTek Laboratories, Logan, UT, USA). Thereafter, the sections were dehydrated in a 1:1 mixture of ethanol and xylene for 5 min and dehydrated in xylene three times (for 5 min each time). Finally, the sections were dried and observed under a microscope (BZ‐X810; KEYENCE).

### Statistical analysis

All statistical analyses were performed using JMP Pro 16 software (SAS Institute Japan, Tokyo, Japan). Data are expressed as mean ± standard deviation. Statistical comparisons between the control group and each experimental group were performed using Student's *t*‐test. Comparisons among three or more groups were performed using the one‐way analysis of variance, followed by Tukey's post‐hoc test. Statistical significance is indicated as *p < 0.05, **p < 0.01 and ***p < 0.001.

## RESULTS

### Effect of GDS‐23 on the expression of genes related to epidermal barrier function and moisture retention

**FIGURE 2:**
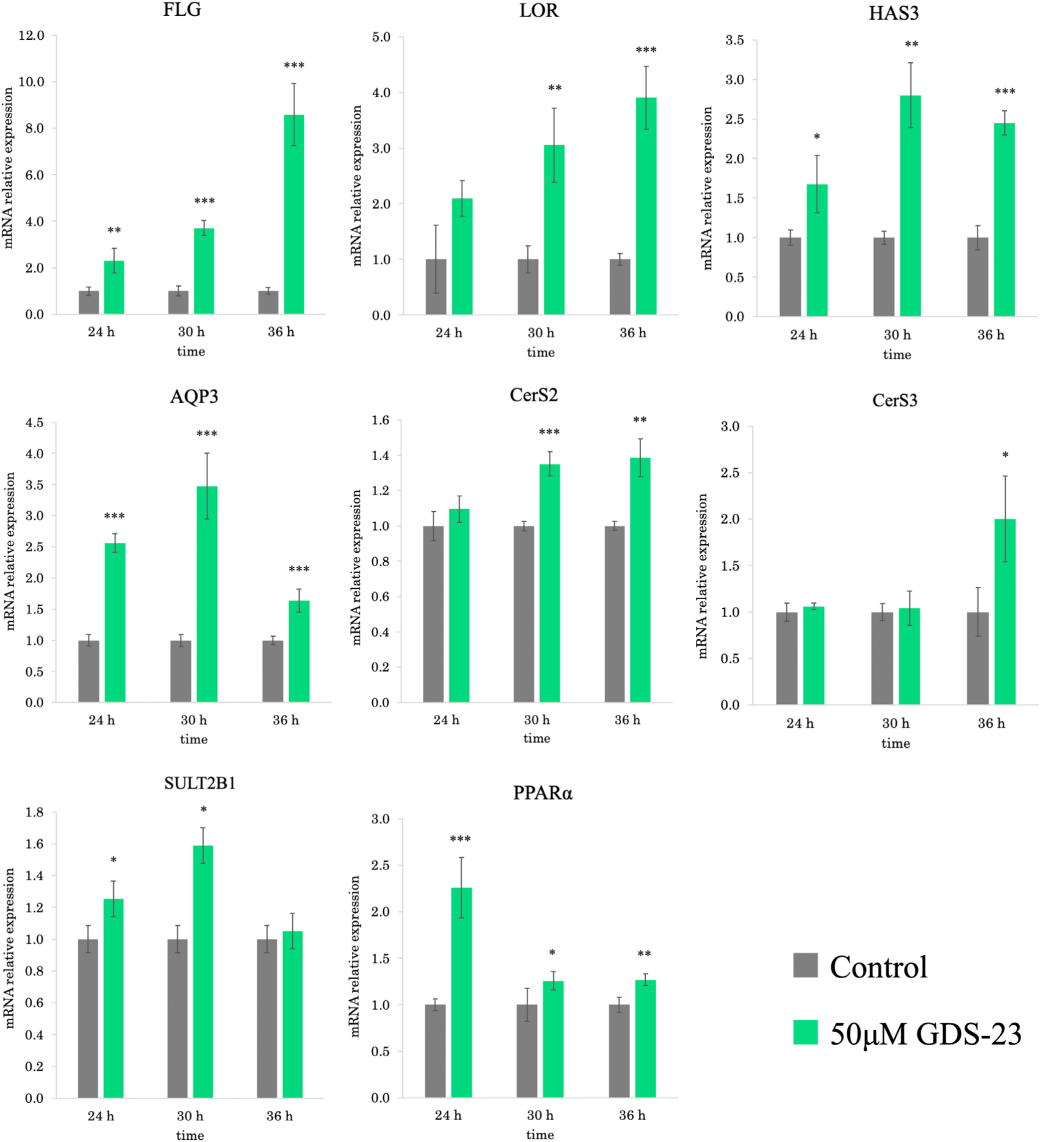
Effects of GDS‐23 on the expression of genes involved in epidermal barrier function and moisture retention. NHEKs were treated with 50 μM GDS‐23 for 24 h, followed by post‐culturing in the medium alone for 6 or 12 h. Cells in the control group were treated with the medium alone. mRNA levels of *FLG*, *LOR*, *HAS3*, *AQP3*, *CerS2*, *CerS3*, *SULT2B1* and *PPARα* were analysed using quantitative PCR. Results are expressed as mean ± standard deviation (*n* = 4). Significance: *p < 0.05, **p < 0.01, ***p < 0.001 vs. control cells (Student's *t*‐test.).

NHEKs were treated with GDS‐23 to examine the effects of GDS‐23 on genes related to epidermal barrier function and moisture retention. In accordance with a previous study that demonstrated Nrf2 activation at around 24 h after GDS‐23 treatment [9], cells were treated with 50 μM GDS‐23 for 24 h, followed by post‐culturing in the medium alone for 6 and 12 h. PCR was performed at 24, 30 and 36 h after GDS‐23 treatment. GDS‐23 treatment significantly increased the mRNA levels of several genes related to epidermal barrier function (*FLG* and *LOR*), moisture retention (*HAS3* and *AQP3*) and lipid synthesis (*CerS2*, *CerS3*, *SULT2B1* and *PPARα*) (Figure 2). In particular, the expression of *FLG* and *LOR* was markedly increased and sustained.

### Role of Nrf2 activation in GDS‐23‐induced upregulation of genes related to epidermal barrier function and moisture retention

**FIGURE 3:**
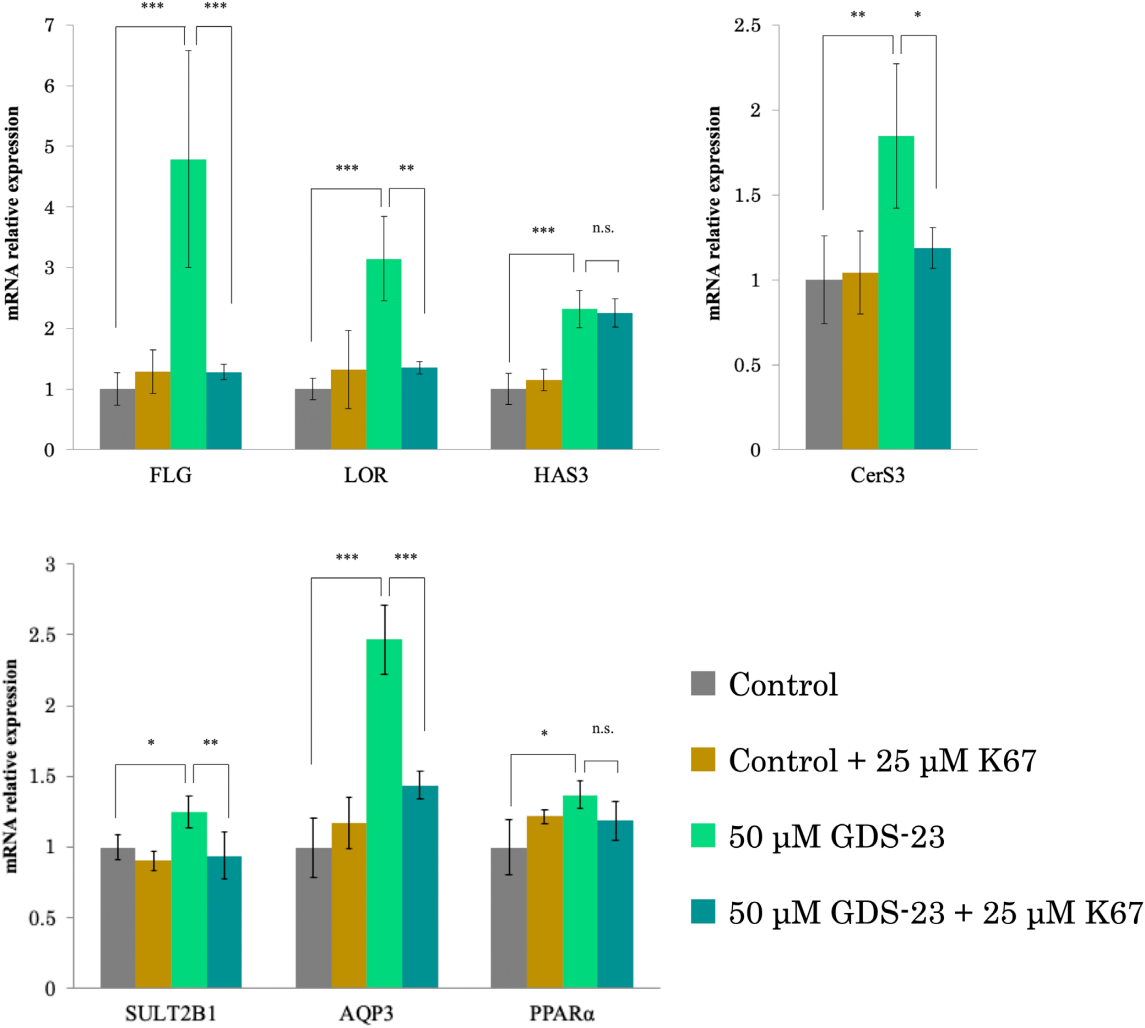
Effect of K67 on GDS‐23‐induced upregulation of genes related to epidermal barrier function and moisture retention. NHEKs were pre‐incubated with 25 μM of K67 or the medium alone for 16 h, and then treated with 50 μM GDS‐23 for 24 h and post‐incubated in the medium alone for 6 or 12 h. Cells in the control group were cultured in the medium alone. mRNA levels of *FLG*, *LOR*, *HAS3*, *AQP3*, *CerS3*, *SULT2B1* and peroxisome *PPARα* were analysed using quantitative PCR. Results are expressed as mean ± standard deviation (*n* = 4). Significance: *p < 0.05, **p < 0.01, ***p < 0.001 (one‐way ANOVA, followed by Tukey's test).

NHEKs were treated with an Nrf2 inhibitor to investigate the role of Nrf2 activation in GDS‐23‐induced upregulation of genes related to epidermal barrier function and moisture retention. Our previous study [9] showed that GDS‐23 activates Nrf2 through the phosphorylation of the autophagy‐related adapter protein p62. Phosphorylated p62 has a high binding affinity for Keap1, allowing Nrf2 to escape capture and translocate to the nucleus, where it induces transcriptional activation of target genes [29]. Therefore, NHEKs were cultured for 16 h in the presence of K67 [30], an inhibitor that blocks the interaction between Keap1 and phosphorylated p62, followed by treatment with GDS‐23 for 24 h and post‐culturing in the medium alone for 6 or 12 h. K67 treatment significantly inhibited GDS‐23‐induced upregulation of *FLG*, *LOR*, *CerS3*, *SULT2B1* and *AQP3* expression. However, K67 treatment did not significantly affect the GDS‐23‐induced upregulation of *HAS3* and *PPARα* mRNA expression (Figure 3).

### Verification of GDS‐23‐induced activation of Nrf2 in the 3D epidermal model

**FIGURE 4:**
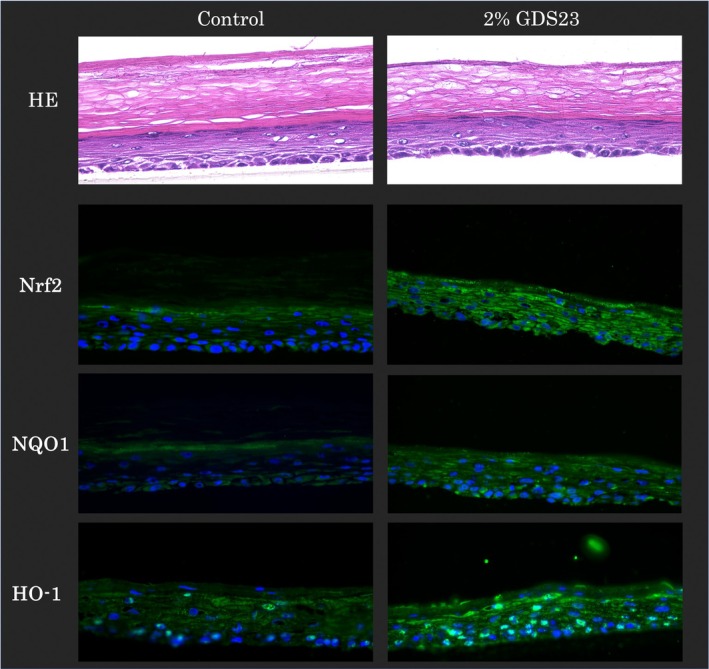
Haematoxylin and eosin (HE) staining and fluorescence immunostaining for antioxidant‐related proteins in a three‐dimensional (3D) epidermal model treated with 2% GDS‐23 solution. Tissues were treated with 2% GDS‐23 solution for 6 h, washed, and incubated for another 18 h without further treatment. The samples were then sectioned, HE stained and immunostained for Nrf2, NQO1 and HO‐1.

GDS‐23 enhances the endogenous antioxidant system in NHEKs through the activation of Nrf2 [9]. Therefore, we examined whether similar results could be obtained in a human epidermal‐like 3D model using fluorescence immunostaining. In the 2D culture system of NHEKs, the test compound comes in direct contact with the cells. Whereas, in the 3D epidermal model, the compound has to permeate the stratum corneum to reach the keratinocytes. Furthermore, the epidermal cells in this model were sustained by a receiver medium (culture medium) underneath, and the medium was considered to infiltrate the cell layers. Therefore, the compound that permeated was assumed to be diluted through mixing with the medium within the model, resulting in a lower actual concentration to which the cells were exposed. Considering the structural differences, we selected a 2% concentration of GDS‐23 (equivalent to approximately 12.2 mM) for the 3D model, based on the findings of previous studies, the absence of cytotoxicity in preliminary experiments and its practical relevance to cosmetic formulations (Figure S1). Treatment of the stratum corneum side of the epidermal model with 2% GDS‐23 increased the fluorescence intensities of Nrf2 and its downstream antioxidant proteins NQO1 and HO‐1 compared with those in the control group (Figure 4). Collectively, these results indicate that GDS‐23 treatment upregulated Nrf2, NQO1 and HO‐1 protein expression in the 3D epidermal model.

### Verification of GDS‐23‐induced upregulation of epidermal barrier function‐related protein expression in the 3D epidermal model

**FIGURE 5:**
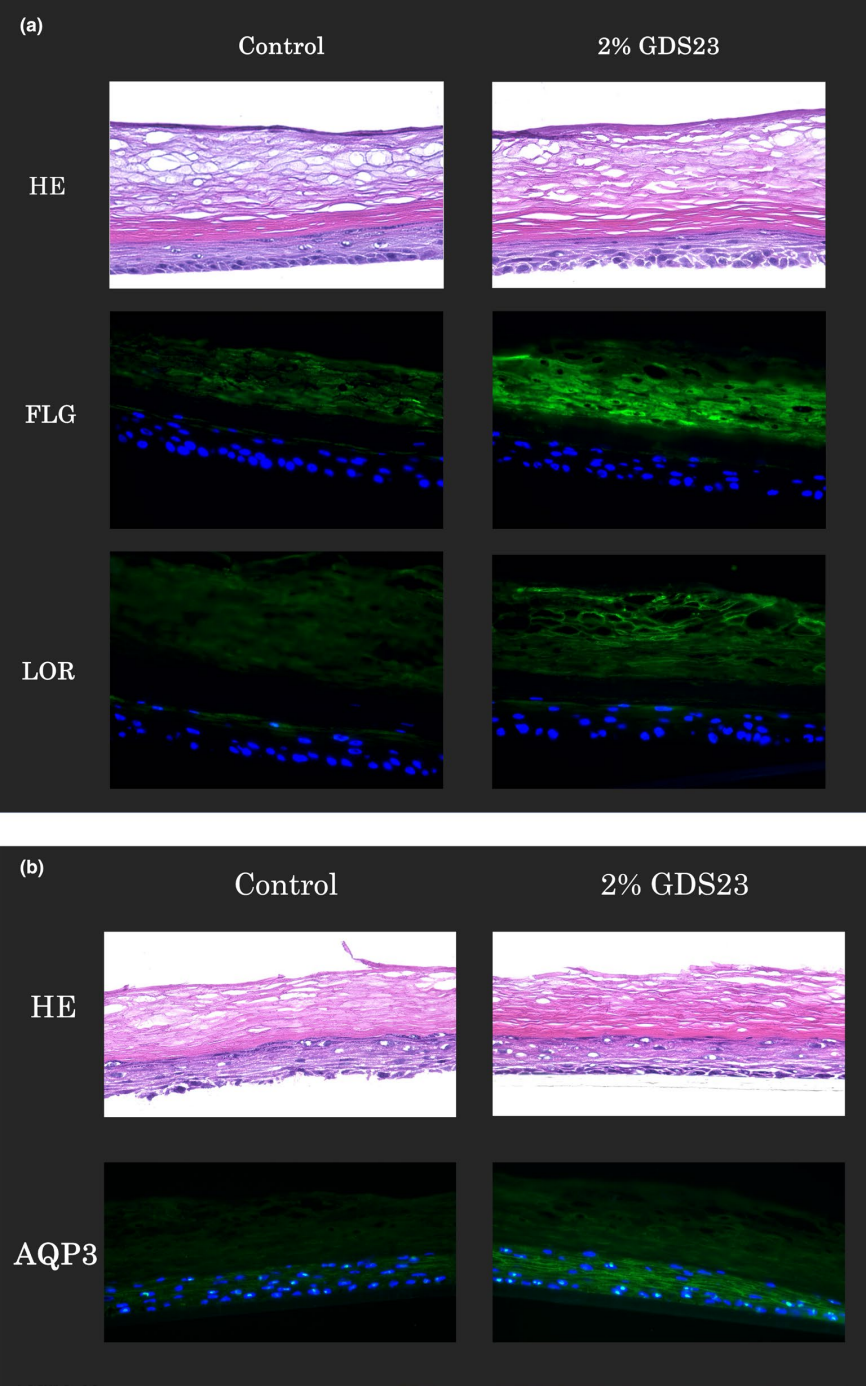
Haematoxylin and eosin (HE) staining and fluorescence immunostaining for proteins related to epidermal barrier function and moisture retention in a three‐dimensional (3D) epidermal model treated with 2% GDS‐23 solution. (a) Tissue sections were stained with HE and immunostained for FLG and LOR after 6 h of treatment with 2% GDS‐23 solution, followed by 7 days of culturing. (b) Tissue sections were stained with HE and immunostained for AQP3 after 6 h of treatment with 2% GDS‐23 solution, followed by an additional 24 h of culturing.

Considering that the expression of FLG, LOR and AQP3 in NHEKs is regulated by Nrf2 activation, we investigated the effects of GDS‐23 on these proteins in a 3D epidermal model. The stratum corneum side of the model was treated with either 2% GDS‐23 or purified water (as a control) for 6 h. After removing the respective treatment agents, the models were further cultured for 7 days to evaluate the expression of FLG and LOR. This post‐culturing was performed to promote proper stratum corneum formation. AQP3 expression was observed after 24 h of post‐culturing following the removal of GDS‐23 from the stratum corneum side. GDS‐23 treatment significantly increased the fluorescence intensity of each target protein in the skin model compared with that in the control group (Figure 5). Overall, these results indicate that GDS‐23 upregulates the expression of FLG, LOR and AQP3 proteins in the 3D epidermal model.

## DISCUSSION

In this study, we investigated the effects of GDS‐23 on epidermal barrier function, moisture retention and skin homeostasis, focusing on changes in the expression of relevant genes. GDS‐23 treatment significantly upregulated the expression of genes involved in stratum corneum barrier formation (*FLG* and *LOR*), intercellular lipid synthesis (*CerS2*, *CerS3* and *SULT2B1*), as well as moisture regulation and retention (*AQP3* and *HAS3*). Additionally, GDS‐23 upregulated *PPARα* expression, which may improve lipid production and promote epidermal barrier function. The skin is constantly exposed to high concentrations of oxygen, ultraviolet rays and chemicals, serving as a crucial barrier against such external harmful stimuli [31]. As part of this protective mechanism, the skin exhibits upregulation of antioxidant factor expression on the outer side of the epidermis, whose induction is regulated via the Nrf2–Keap1 signalling pathway [10, 32, 33].

FLG, which is crucial to stratum corneum barrier formation, is a histidine‐rich protein that is broken down into hygroscopic NMFs during the final stage of keratinization. Notably, histidine and its derivatives, such as urocanic acid, are products of FLG degradation and play a role in epidermal pH regulation and antioxidant activity [34, 35]. LOR, also essential for stratum corneum barrier formation and a major component of the CE, contains several cysteine residues and is involved in the antioxidant activity of the epidermis [10, 36]. Nrf2 activation reportedly upregulates both FLG and LOR expression [37, 38]. Moreover, a previous study showed that GDS‐23 treatment activated Nrf2 in NHEKs within 12 to 24 h [9]. Consequently, our findings suggest that GDS‐23‐induced upregulation of FLG and LOR expression after 24 h of treatment is mediated by Nrf2 activation. Furthermore, the suppression of GDS‐23‐induced upregulation of FLG and LOR expression by the Nrf2 inhibitor K67 [30] suggests that the Nrf2 pathway is at least partially involved.

Partial or complete downregulation of profilaggrin and FLG expression leads to impaired stratum corneum formation and decreased barrier function, thereby facilitating transdermal penetration of allergens [39]. Similarly, a reduction in the level or loss of LOR diminishes barrier permeability, leading to increased moisture loss and enhanced transdermal penetration of foreign substances. In cases of LOR deficiency, small proline‐rich proteins and other molecules are reportedly induced via the Nrf2 pathway as a compensatory response [40]. However, these proteins cannot fully replicate the structural and biochemical functions of LOR [41]. Mutations or deficiencies in FLG and LOR are associated with impaired skin function and the onset of various skin diseases, including atopic dermatitis and psoriasis [28, 42]. Therefore, GDS‐23‐induced upregulation of FLG and LOR expression may represent a novel therapeutic option for maintaining skin homeostasis and treating skin diseases in the future.

In the present study, GDS‐23 treatment upregulated the expression of *CerS2*, *CerS3* and *SULT2B1*, which encode enzymes involved in the synthesis of ceramides and sulfated cholesterol, key components of the intercellular lipids of the stratum corneum barrier. The CerS family of ceramide synthases is involved in the synthesis of ceramides using long‐chain bases and acyl‐CoA as substrates [43]. Notably, CerS2 synthesizes C20–C26 ceramides, whereas CerS3 synthesizes C22–C36 long‐chain ceramides, which are important for skin barrier function [44, 45]. SULT2B1 is a member of the hydroxysteroid sulfotransferase enzyme family and has two isoforms, SULT2B1a and SULT2B1b, with SULT2B1b synthesizing sulfated cholesterol from cholesterol. Importantly, the SULT2B1b isoform is specifically expressed in the skin. Therefore, the gene expression observed in this study is likely *SULT2B1b* expression [46, 47]. Previous studies have shown that *PPAR*s upregulate the mRNA expression of *CerS2*, *CerS3* and *SULT2B1* [48, 49, 50]. Additionally, GDS‐23 treatment reportedly upregulates *PPARγ* [9] and *PPARα* mRNA expression. Collectively, these results suggest that *PPARs* may be involved in the GDS‐23‐induced upregulation of *CerS2*, *CerS3* and *SULT2B1* mRNA expression. On the contrary, the significant suppression of GDS‐23‐induced *CerS3* and *SULT2B1* expression by Nrf2 inhibition indicates the regulation of expression of these genes, at least partially, by the Nrf2 pathway; however, further investigation is needed to clarify the underlying mechanisms. Although there are reports that *PPAR* expression is regulated by Nrf2 [51, 52], it is also conceivable that GDS‐23 acts directly as an agonist of PPARα. However, further studies are necessary to elucidate the detailed mechanisms underlying the GDS‐23‐induced upregulation of *CerS3*, *SULT2B1* and *PPARα* mRNA expression.

Downregulation of *CerS2*, *CerS3* and *SULT2B1* expression can induce a decrease in intercellular lipids, resulting in the loss of the lamellar structure and the impairment of the permeability barrier [15, 44, 47]. These enzymes play a crucial role in maintaining the homeostasis of the epidermal barrier. Particularly, ultra‐long‐chain ceramides are vital for the maintenance of epidermal lipid homeostasis [53]. Therefore, the loss of ceramides and sulfated cholesterol owing to deficiencies in CerS2, CerS3 and SULT2B1 is implicated in not only the reduction of barrier function but also the pathogenesis of skin diseases such as psoriasis and ichthyosis. Conclusively, GDS‐23‐induced upregulation of various epidermal barrier function‐related genes, including *FLG* and *LOR*, may be beneficial for normalising epidermal barrier functions and could have future therapeutic applications in skin diseases.

Here, GDS‐23 treatment upregulated the mRNA expression of the moisture‐related factors *AQP3* and *HAS3*, which are involved in maintaining skin homeostasis. AQP3, a transmembrane protein in the epidermis, transports not only water but also glycerol, hydrating the epidermis and promoting wound healing in cases of trauma or burns [54]. HAS3 plays a crucial role in biological functions, such as moisture retention, tissue repair and regeneration, scaffolding formation, as well as cell proliferation and migration during wound healing [21, 22, 55]. These findings suggest a potential role of GDS‐23 in supporting wound healing in damaged tissues. Additionally, inhibition of Nrf2 activation significantly suppressed the GDS‐23‐induced upregulation of *AQP3* mRNA expression. Meanwhile, K67 treatment did not affect GDS‐23‐induced upregulation of *HAS3* expression. HAS3 expression has been reported to be regulated via PPARα activation [55, 56], suggesting partial involvement of PPARα; however, further investigation is required.

To investigate the pharmacological effects of GDS‐23 under conditions similar to actual human skin conditions, we conducted experiments using a 3D epidermal model. Specifically, we utilized LabCyte EPI‐MODEL [57], which replicates the characteristics of the actual human epidermis, including the structure of the epidermal layer with the stratum corneum and the distribution of epidermal differentiation markers. In our preliminary study, continuous 24 h exposure of the stratum corneum side to the test solution caused deterioration of the stratum corneum structure in the 3D model, likely due to excessive hydration leading to a swollen and softened surface. Therefore, the treatment duration was limited to 6 h to preserve tissue integrity. This choice was further supported by preliminary data from a calcein sodium‐based transdermal penetration study showing that absorption can occur within 6 h (Figure S2). Furthermore, from the perspective of replicating the conditions of a previous study [9], the protocol used in this study—applying GDS‐23 for 6 h followed by sample removal and post‐incubation—appears to be consistent with prior protocols, as GDS‐23 is retained within the skin model. Although increased calcein sodium permeability was observed, it does not necessarily indicate impairment of barrier function. Observations of HE‐stained sections in this study (Figures 4 and 5) indicated that the structure of the stratum corneum was preserved. In addition, niosomes derived from GDS‐23 were composed of non‐ionic surfactants, were amphiphilic, and had a particle diameter of approximately 100–200 nm (Figure 1, Figure S3). These multilamellar niosomes have been reported to deliver their contents to deeper epidermal layers without disrupting the stratum corneum structure, by partially fusing with lipid layers or passing through the intercellular lipid route, depending on physicochemical properties such as particle size and membrane fluidity [58]. We also hypothesize that the niosomes used in this study experience partial disassembly of the outer lamellar structures during passage, but can still reach viable keratinocytes while retaining their structural integrity. Further investigation is required to determine whether the niosomes composed of GDS‐23 retain their complete structure during transdermal penetration. Therefore, we examined the expression of proteins related to the antioxidant system, stratum corneum formation and moisture retention. Considering that GDS‐23 treatment induces Nrf2 activation in epidermal keratinocytes [9], we verified the expression of Nrf2 and its downstream antioxidant proteins (NQO1 and HO‐1) in the 3D epidermal model. GDS‐23 treatment upregulated the expression of Nrf2 and the antioxidant proteins NQO1 and HO‐1 in the 3D model. Under normal conditions, Nrf2 forms a complex with Keap1 in the cytoplasm and is ubiquitinated and degraded by Keap1 [59]. Typically, Nrf2 is released from Keap1 and translocated to the nucleus to activate the transcription of target genes in cells under oxidative stress or stimulation with electrophilic substances [60, 61]. Moreover, phosphorylated p62 has a strong affinity for Keap1, which facilitates the stabilization and release of Nrf2 [29]. In a previous study, GDS‐23 treatment induced p62 phosphorylation in NHEKs, which promoted Nrf2 activation and enhanced the expression of various antioxidant‐related proteins [9]. These findings suggest that GDS‐23 may stabilize Nrf2 in a p62‐dependent manner in the epidermal model, leading to increased Nrf2 expression. This stabilization could enhance the transcriptional activation of downstream antioxidant enzymes such as NQO1 and HO‐1, resulting in their elevated levels.

We also examined the effects of GDS‐23 on the expression of epidermal barrier function‐related and moisture‐related proteins in the 3D epidermal model. FLG and LOR are expressed in the granular layer during epidermal cell differentiation and are predominantly localized to the stratum corneum cells and their periphery [19, 39]. Consistent with the results observed in NHEKs, GDS‐23 treatment upregulated the expression of proteins related to epidermal barrier function (FLG and LOR) and moisture retention (AQP3) in the 3D epidermal model. AQP3 is localized to the cell membrane of keratinocytes, particularly in layers below the granular layer [24]. Similarly, AQP3 expression was detected near living cells in the epidermis in the present study. Overall, GDS‐23‐induced activation of Nrf2 and upregulation of FLG, LOR and AQP3 expression in both NHKE cells and the 3D epidermal model suggests that GDS‐23 may enhance skin homeostasis.

The above findings indicate that GDS‐23 may help maintain skin homeostasis by modulating the expression of molecular factors associated with epidermal barrier and moisture retention via Nrf2 activation. Additionally, GDS‐23 treatment increased the expression of molecular markers associated with endogenous antioxidant function, epidermal barrier function and moisture retention in the 3D epidermal model. These results suggest that GDS‐23 may be beneficial in the management of skin conditions such as atopic dermatitis, psoriasis and ichthyosis. As a member of the PEG lipid family, GDS‐23 has potential application in multifunctional DDS as a therapeutic agent for improving transdermal drug absorption, enhancing endogenous antioxidant function, as well as strengthening epidermal barrier function and moisture retention to maintain skin homeostasis and facilitate wound healing. In summary, this study provides valuable insights into the potential applications of GDS‐23 in skin care, the management of skin conditions and development of cosmetic formulations.

## CONFLICT OF INTEREST STATEMENT

TM is an employee of Beverly Glen Laboratories, Inc. BCK is the Chief Science Officer of Beverly Glen Laboratories, Inc. SN, TA and SN serve as members of a joint research project between Beverly Glen Laboratories, Inc. and Showa Medical University. The authors declare that they have no known competing financial interests or personal relationships that could have appeared to influence the work reported in this paper.

## Supporting information


Figure S1

Figure S2

Figure S3

## Data Availability

The data that support the findings of this study are available from the corresponding author upon reasonable request.
