# Supplementary figures and images for: PEG‐23 glyceryl distearate, a multifunctional skin‐supporting material, upregulates the expression of factors associated with epidermal barrier and hydration
Source: Int J Cosmet Sci. 2025 Jul 10;47(6):1056–69. doi: 10.1111/ics.70005 (PMC12666733; doi:10.1111/ics.70005)

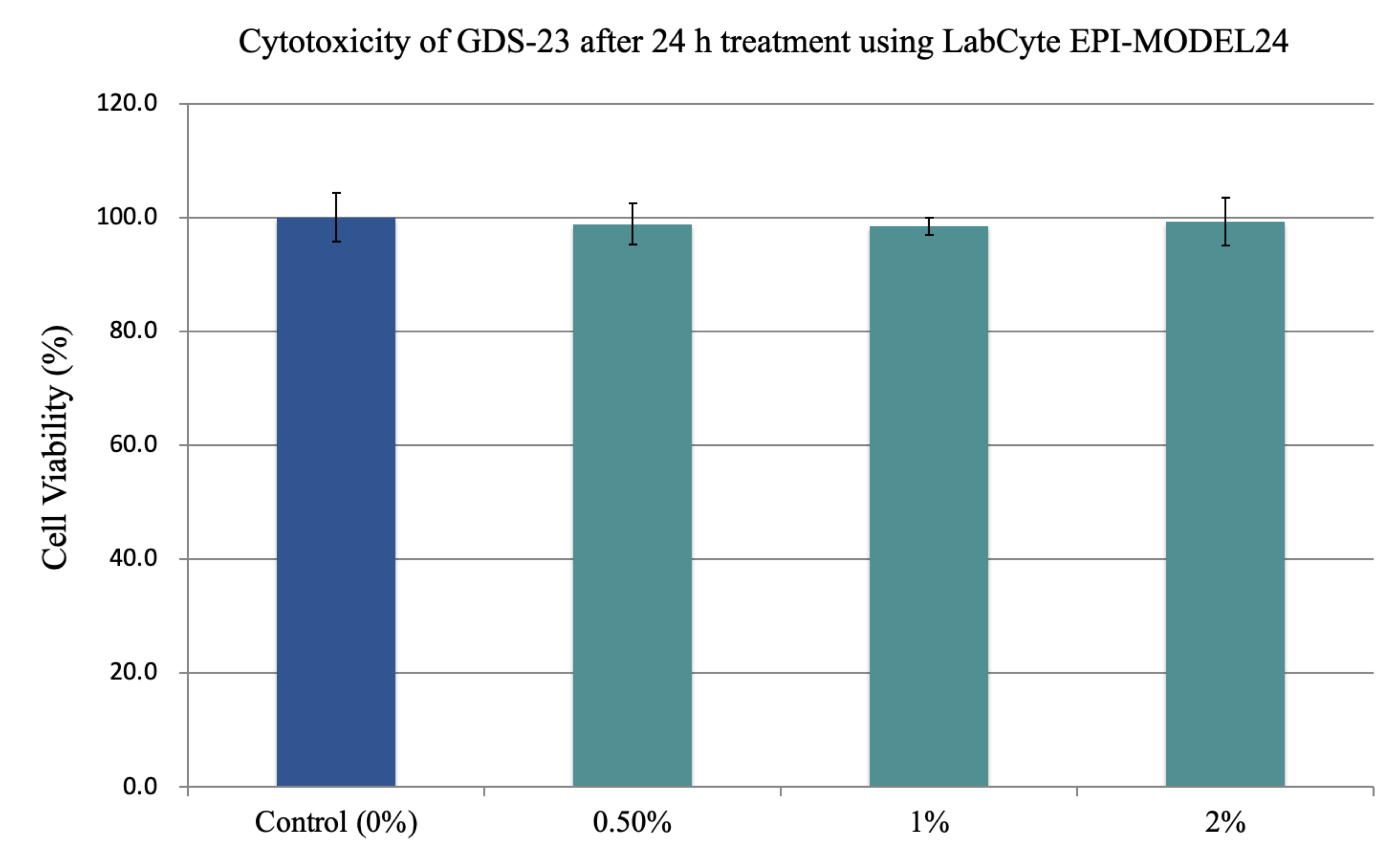

Supplement: Supplementary file 1 — Figure S1 [file ICS-47-1056-s003.tiff]

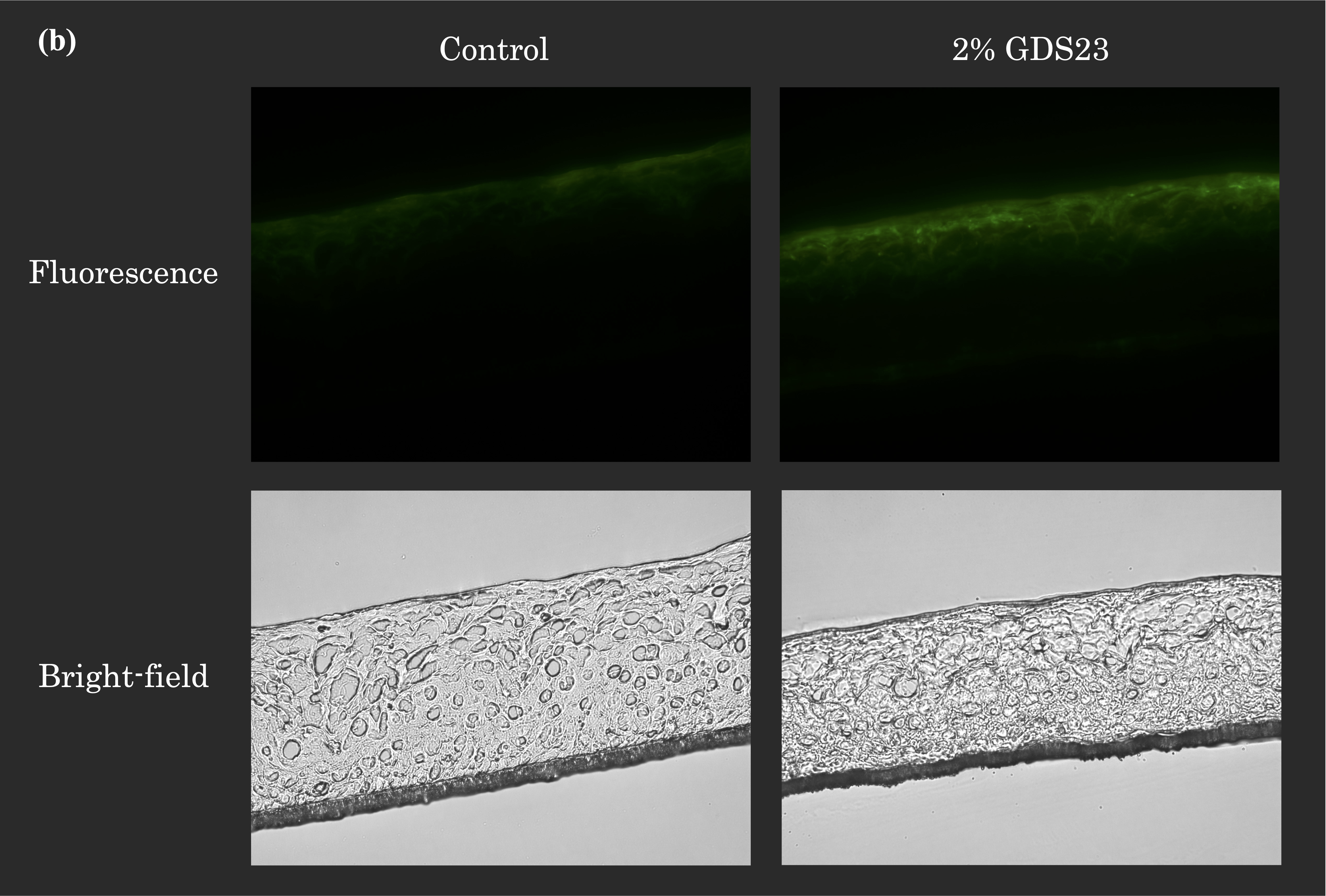

Supplement: Supplementary file 2 — Figure S2 [file ICS-47-1056-s002.zip › ics70005-sup-0003-FigureS2b.tiff]

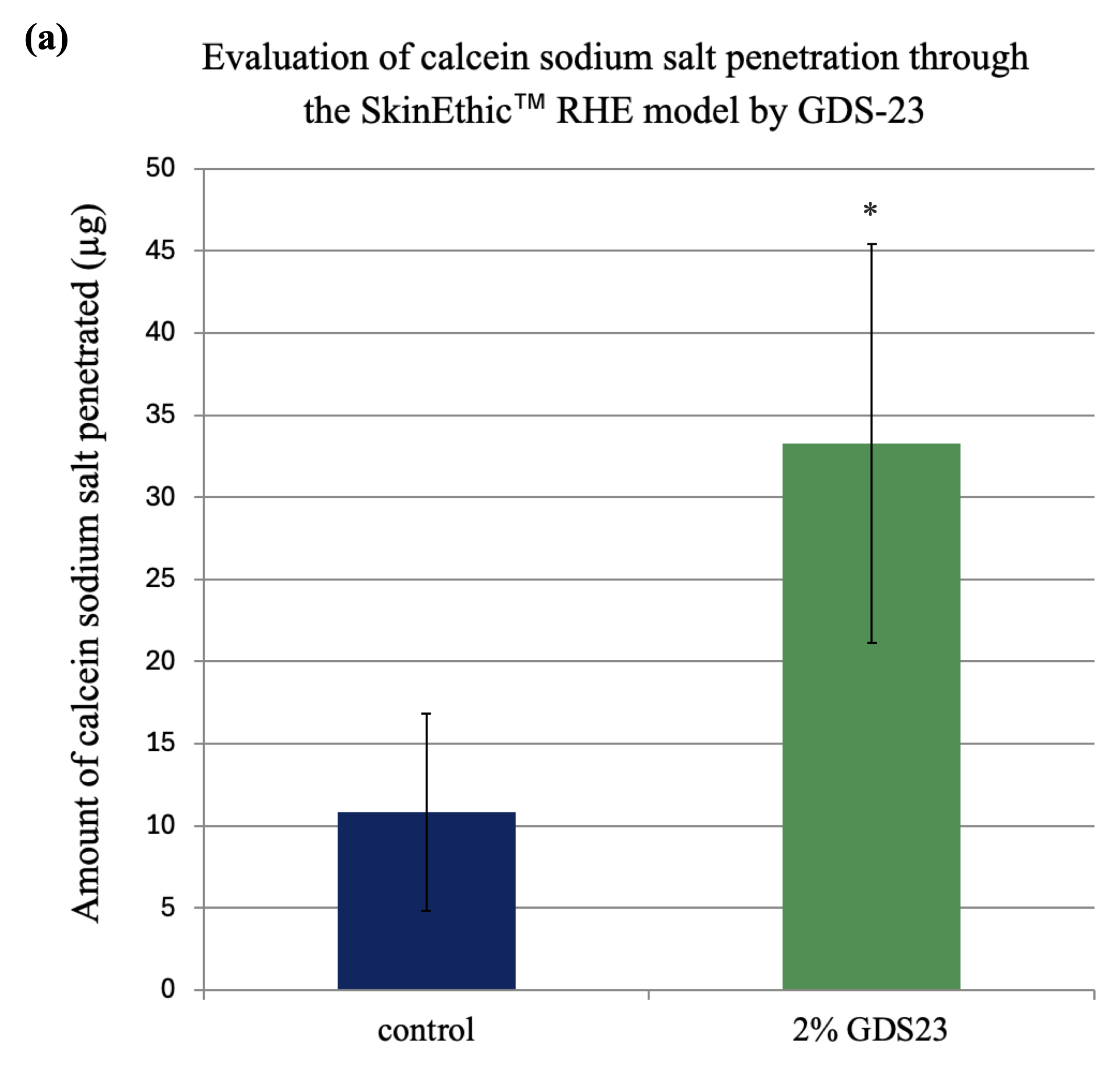

Supplement: Supplementary file 2 — Figure S2 [file ICS-47-1056-s002.zip › ics70005-sup-0002-FigureS2a.tiff]

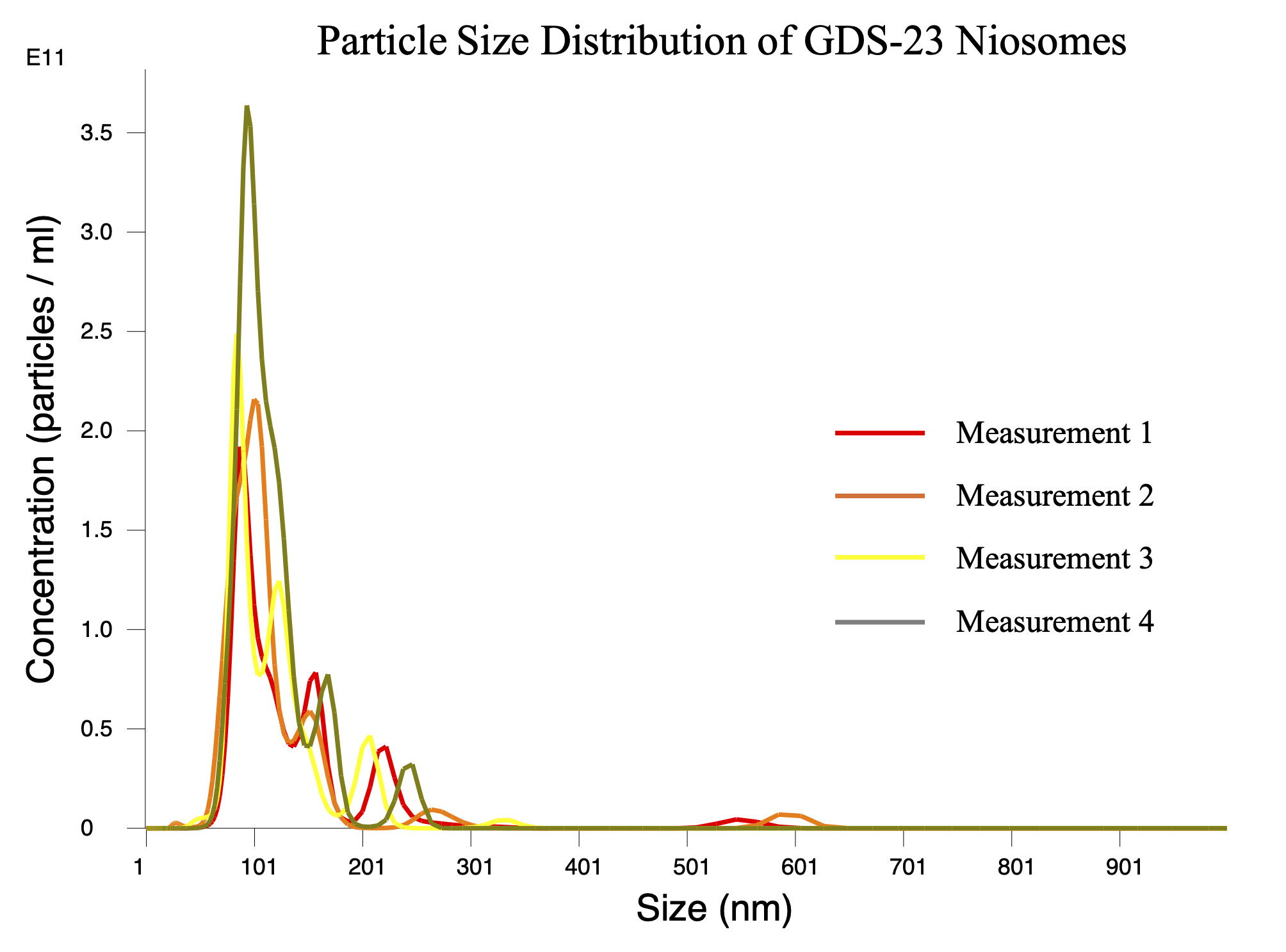

Supplement: Supplementary file 3 — Figure S3 [file ICS-47-1056-s001.tiff]
